# *Thais savignyi* tissue extract: bioactivity, chemical composition, and molecular docking

**DOI:** 10.1080/13880209.2022.2123940

**Published:** 2022-10-06

**Authors:** Mohamed R. Habib, Ahmed A. Hamed, Rasha E. M. Ali, Khaled M. Zayed, Rasha M. Gad El-Karim, Rehab Sabour, Hanaa M. Abu El-Einin, Mosad A. Ghareeb

**Affiliations:** a Medical Malacology Department, Theodor Bilharz Research Institute, Giza, Egypt; b Microbial Chemistry Department, National Research Center, Giza, Egypt; c Pharmaceutical Medicinal Chemistry and Drug Design Department, Faculty of Pharmacy (Girls), Al-Azhar University, Cairo, Egypt; d Medicinal Chemistry Department, Theodor Bilharz Research Institute, Giza, Egypt

**Keywords:** Antioxidant, antimicrobial, cytotoxicity, bioactive compounds, marine snails

## Abstract

**Context:**

*Thais savignyi* Deshayes (Muricidae) is widely distributed in the Red Sea. Its abundance and the history of Muricidae in traditional medicine make it a tempting target for investigation.

**Objective:**

To investigate the chemical profile and biological activities of *T. savignyi* tissue extracts.

**Materials and methods:**

Methanol, ethanol, acetone, and ethyl acetate extracts from *T. savignyi* tissue were compared in their antioxidant by total antioxidant capacity, DPPH free radical scavenging, and total phenolic content. In addition, the antimicrobial, and antibiofilm properties (at 250 µg/mL) of the extracts were tested against *Escherichia coli*, *Pseudomonas aeruginosa*, *Proteus vulgaris*, *Klebsiella pneumoniae*, *Staphylococcus aureus*, and *Candida albicans*. The antioxidant extract with greatest activity was assessed for cytotoxicity (range 0.4–100 µg/mL) against 3 human cancer cell lines (UO-31, A549 and A431), and its chemical composition was investigated using GC-MS. Moreover, docking simulation was performed to predict its constituents’ binding modes/scores to the active sites of thymidylate kinase.

**Results:**

The ethyl acetate extract (*Ts*-EtOAc) showed the highest total antioxidant capacity (551.33 mg AAE/g dry weight), total phenolics (254.46 mg GAE/g dry weight), and DPPH scavenging (IC_50_= 24.0 µg/mL). *Ts*-EtOAc exhibited strong antibacterial (MIC: 3.9 µg/mL against *K. pneumoniae*), antibiofilm (MIC: 7.81 µg/mL against *S. aureus*), and antifungal (MIC: 3.9 µg/mL against *C. albicans*) activities and considerable cytotoxicity against cancer cells (UO-31: IC_50_= 19.96 ± 0.93, A549: IC_50_= 25.04 ± 1.15 μg/mL). GC-MS identified multiple bioactive metabolites in *Ts*-EtOAc extract belonging to miscellaneous chemical classes. Molecular docking studies revealed that the constituents of *Ts*-EtOAc have antibacterial potential.

**Discussion and conclusions:**

*T. savignyi* extract has considerable antimicrobial and cytotoxic activities. Further studies are needed to isolate the active constituents of this snail for comprehensive drug discovery tests.

## Introduction

New efficient drugs and bioactive chemicals for treating human disorders such as cancer, inflammation, and microbial infection are sorely needed (Newman and Cragg 2020; Atanasov et al. 2021). The marine ecosystem is characterised by the high biodiversity of mollusc species, with more than 50,000 identified species (Benkendorff 2010), making it an excellent source for discovering novel bioactive compounds and pharmaceuticals (de Vries and Beart 1995).

Molluscs are notable for surviving in a challenging marine environment teeming with harmful viruses and bacteria. At the same time, they lack acquired immunity and rely only on innate immunity with alternate strategies for defending themselves against predators and successfully healing wounds in the presence of various microorganisms (Bachere et al. 1995; Derby et al. 2007). This protection is mediated by the production of secondary metabolites, which are also used in communication and predatory behaviours by molluscs (Cimino and Sodano 1993; Craig 2000; Kanda et al. 2003; Cummins et al. 2006; Hooper et al. 2007). Indeed, many secondary metabolites have been isolated from various marine mollusc species (Benkendorff 2010). Although not all molluscan natural products were pharmacologically tested, some have demonstrated considerable biological activity, including antibacterial and cytotoxic activities (Odeleye et al. 2019).

Screening for cytotoxic activities has led to several molluscan products being investigated as anticancer treatments, while some molluscan natural products have shown promising anti-coagulant, immune-modulating, antioxidant, anti-inflammatory, and antihypertensive properties (Jimeno et al. 2004; Senthilkumar and Kim 2013; Ahmad et al. 2018). Thousands of bioactive compounds have been isolated from marine molluscs. These include peptides, sterols, terpenes, polypropinate, nitrogenous compounds, macrolides, fatty acid derivatives, alkaloids, and other miscellaneous compounds (Blunt et al. 2006; Chakraborty and Joy 2020). Some of these have been the subject of clinical trials, such as dolastatin 10 from the opisthobranch *Dolabella Auricularia* Lightfoot (Aplysiidae), aplyronine A from the sea hare *Aplysia kurodai* Baba (Aplysiidae), aplysistatin from *A. angasi* G.B. Sowerby II (Aplysiidae), and kahalalide F from the sacoglossan *Elysia rufescens* Pease (Plakobranchidae) (Proksch et al. 2002). Two well-known natural molluscan products have been used in the development of novel drugs: ziconotide, isolated from the venom of the cone snail *Conus magus* L. (Conidae), which is used as an analgesic to treat chronic pains (Schroeder et al. 2004; Mayer et al. 2010), and brentuximab vedotin, derived from dolastatin 10 from *D. auricularia* which is used for the treatment of Hodgkin lymphoma and systemic anaplastic large cell lymphoma (Mayer et al. 2010). Both drugs are approved by the US Food and Drug Administration (FDA) (Cheung et al. 2015).

*Thais savignyi* Deshayes (Muricidae) is a carnivorous gastropod snail usually found attached to rocks. Muricid gastropods have a history of use in traditional medicine, and this snail has been the subject of numerous investigations related to its distribution, identification, and molecular phylogeny (Hayashi 2005; Li et al. 2010; Benkendorff et al. 2015). *T. savignyi* is distributed along the intertidal zones of the Red Sea (Abu El-Einin et al. 2021). The present study investigates the antioxidant, antimicrobial, and cytotoxic activities of crude tissue extract from *T. savignyi* and its chemical composition. In addition, *in silico* docking studies were performed to identify the possible binding interactions of the identified compounds in *T. savignyi* extract with thymidylate kinase, a key enzyme in bacterial DNA biosynthesis, bacterial survival, and one of the attractive therapeutic targets for the development of new antibacterial agents (Choi et al. 2012; Keating et al. 2012).

## Materials and methods

### Samples collection

*Thais savignyi* was collected from the Ain El-Sokhna region on the Red Sea, Egypt, during October 2020. Details of sample collection and identification are described in Abu El-Einin et al. (2021). Snails were cleaned using distilled water, the shells were broken, and the soft parts were dissected. The freshly collected tissues were divided into four 50 g portions. For organic extraction, each portion was blended and soaked separately in a volume of 500 mL each of methanol (*Ts*-MeOH), ethanol (*Ts*-EtOH), acetone (*Ts*-Acetone), and ethyl acetate (*Ts*-EtOAc), and kept in conical flasks for 3 days in a dark place. The solutions were filtered through Whatman filter paper (No. 1) and concentrated using a rotary evaporator (Buchi, Switzerland). The obtained extracts were completely air-dried, to remove any solvent residues, and stored at 4 °C until used in the analysis.

### DPPH free radical scavenging activity

The free radical scavenging activity of the four snail extracts was determined based on the scavenging activity of 1,1-diphenyl-2-picrylhydrazyl (DPPH) (Sigma-Aldrich, Germany) according to Brand-Williams et al. (1995) and Ullah et al. (2018). Various concentrations from each extract (50–500 µg/mL) were prepared in methanol and 0.1 mL from each concentration was added to 3.9 mL of 6 × 10^−5 ^mol/L DPPH in methanol. The mixture was shaken vigorously. A blank sample containing the same volume of methanol and DPPH was prepared to serve as the control. Ascorbic acid (Merck, Germany) was used as a standard. After 30 min of incubation in the dark, the absorbance of the control and test samples was measured at 517 nm. The experiment was carried out in triplicate. The radical scavenging activity was expressed as IC_50_ values, which indicate the concentration of sample required to scavenge 50% DPPH free radicals (Braca et al. 2003; Gülçın et al. 2007). IC_50_ was calculated according to the following equation:

(1) $$\%\;\text{DPPH}\;\text{radical}\;\text{scavenging}\;=\;\frac{\text{absorbance}\;\text{of}\;\text{control}\;-\;\text{absorbance}\;\text{of}\;\text{test}\;\text{sample}}{\text{absorbance}\;\text{of}\;\text{control}}\text{ × }100$$

### Total antioxidant capacity (TAC) by phosphomolybdenum method

The total antioxidant capacity of each extract was evaluated according to the phosphomolybdenum method using ascorbic acid as a standard (Prieto et al. 1999). Briefly, 0.5 mL of each sample extract and ascorbic acid (200 µg/mL) in methanol were combined in dry tubes with 5 mL of molybdate reagent solution (1 mL of 0.6 M sulphuric acid, 28 mM sodium phosphate and 4 mM ammonium molybdate were added to 20 mL of distilled water and the mixture was made up to 50 mL using distilled water). The reaction mixture tubes were then firmly capped and incubated in a thermal block at 95 °C for 90 min. Following incubation, samples were left to cool at room temperature for 20–30 min, and the absorbance was measured at 695 nm using a spectrophotometer against a blank sample containing all reagents, solvents and methanol (0.5 mL) instead of sample extract. All experiments were carried out in triplicate. The total antioxidant activity was expressed as the number of ascorbic acid (AAE) gram equivalents.

### Total phenolic content (TPC)

The total phenolic content of each extract was estimated using the Folin-Ciocalteu’s reagent according to Singleton and Rossi (1965). A volume of 0.1 mL from each extract in methanol (200 µg/mL) was mixed with 0.5 mL of the Folin-Ciocalteu’s reagent and 1.5 mL of sodium carbonate (20 g/100 mL water). A final volume of 10 mL was achieved by adding distilled water. Following incubation for 2 h at room temperature, the absorbance of the mixture was measured at 765 nm using a spectrophotometer (UVmini-1240, Shimadzu Corp., Kyoto, Japan). Gallic acid was used as a standard for the calibration. All tests were performed in triplicate. The total phenolic content was expressed as mg of gallic acid equivalent (GAE) per g of extract (Alhakmani et al. 2013; Ghareeb et al. 2014).

### Antibacterial and antifungal activity

Antimicrobial activity for each extract of *T. savignyi* was assessed against six human pathogenic bacterial strains (four Gram-negative bacteria; *Escherichia coli* ATCC 25955, *Pseudomonas aeruginosa* ATCC 10145, *Proteus vulgaris*, and *Klebsiella pneumoniae*, and one Gram-positive bacteria; *Staphylococcus aureus* NRRL B-767) and one pathogenic fungal strain (the yeast *Candida albicans* ATCC 10231) according to the National Committee for Clinical Laboratory Standards (Wayne 2002). The minimum inhibitory concentration (MIC) was determined and Ciprofloxacin (control drug) was used for comparison as described by Hamed et al. (2020). Each strain of bacteria and fungi was cultured overnight in lysogeny broth (LB) medium at 30 °C. The culture was then adjusted to an optical density (OD) of 0.6 at a wavelength of 600 nm. Sample extracts were dissolved in methanol and 10 µL of test extracts (250 µg/mL) were added to a sterile 96-well flat polystyrene plate containing 80 µL of LB. Finally, 10 µL of bacterial culture suspension (log phase), were added to obtain a final volume of 100 µL/well. Wells containing only LB medium with inoculum served as a blank. Microplates were incubated overnight at 37 °C. After incubation, 20 μL of 3-(4,5-dimethyl-thiazol-2-yl)-2,5-diphenyl-tetrazolium bromide (MTT) at a concentration of 1 mg/mL was prepared in water, filtered and added to each well, and the plates were stirred for 20 min in the dark. The viable bacteria were detected by the change of the yellow MTT colour to purple. The absorbance was measured using a Spectrostar Nano Microplate Reader (BMG LABTECH GmbH, Allmendgrun, Germany).

### Biofilm inhibition assay

The biofilm inhibitory activity of sample extracts against four clinical microbes compromising *S. aureus, Bacillus subtilis*, *P. aeruginosa,* and *E. coli* was measured using a sterile 96-well polystyrene (flat bottom) microtitre tissue culture plate as described by Kalishwaralal et al. (2010) with modification by Balasamy et al. (2019). Briefly, bacterial strains were cultured overnight in LB broth medium, and 10 µL of this culture was added to 180 µL of LB broth, followed by 10 µL (final concentration of 250 g/mL) of samples and control (without test sample). The plates were incubated at 37 °C for 24 h. Following incubation, the content of each well was removed and washed twice with 200 μL phosphate buffer saline (PBS, pH 7.2) to remove any floating bacteria, and the wells were left to dry. After fixation with 2% sodium acetate, the test samples were stained with 200 μL crystal violet solution (0.1% w/v) for 1 h. The excess dyes were removed with sterile water, and the wells were washed with PBS and left to dry. Further, the stain was solubilised in 95% ethanol and biofilm formation was quantified by measuring the absorbance at OD at 570 nm wavelength using a Spectrostar Nano Microplate Reader (BMG LABTECH GmbH, Allmendgrun, Germany). The percent of biofilm inhibition was calculated using the following equation:

(2) $$\%\;\text{biofilm}\;\text{inhibition}\;=\;\frac{\text{OD}\;\text{of}\;\text{control}\;-\;\text{OD}\;\text{of}\;\text{the}\;\text{tested}\;\text{sample}}{\text{OD}\;\text{of}\;\text{control}}\text{ × }100$$

### Cytotoxicity assay

Cell lines for human kidney renal cell carcinoma UO-31, adenocarcinomic human alveolar basal epithelial cells A549 and human epidermoid carcinoma A431 were obtained from ATCC via the holding company for biological products and vaccines (VACSERA), Cairo, Egypt. Staurosporine was used as a positive control for comparison. The cell lines were freshly cultivated as monolayers in RPMI-1640 medium (Sigma Co., St. Louis, MO, USA), supplemented with 10% heat-inactivated fetal bovine serum (GIBCO, UK), 1% glutamine, 100 units/mL penicillin and 100 µg/mL streptomycin, and incubated at 37 °C in a 5% CO_2_ incubator. The cytotoxicity of the tested extract was evaluated using a colorimetric MTT assay. Cancer cell lines (1 × 10^4^ cells/mL) were seeded in 96 flat-bottom well plates for screening. Different concentrations of *Ts*-EtOAc extract (0.4–100 µg/mL) were added to the cultures (in triplicates) and incubated for 24 h in 5% CO_2_ incubator. After 24 h, the cells were washed with PBS, mixed with 20 µL of MTT (Sigma Co., St. Louis, MO, USA) at a 5 mg/mL concentration, and subsequently incubated for 4 h at 37 °C in a 5% CO_2_ incubator. The purple formazan crystals formed were washed with 100 µL of DMSO, and each well’s optical density was observed. The colorimetric assay is measured and recorded at an absorbance of 570 nm using a plate reader (EXL 800, USA). The relative cell viability expressed as a percentage was calculated as follows:

(3) $$\%\;\text{cell}\;\text{viability}\;=\;\frac{\text{absorbance}\;\text{of}\;\text{treated}\;\text{samples}\text{ (A}570)}{\text{absorbance}\;\text{of}\;\text{untreated}\;\text{samples}\;(\text{control};\;A570)}\text{ × }100$$

### Gas chromatography-mass spectrometry (GC-MS) analysis

GC-MS investigation of *Ts*-EtOAc extract was performed utilising a Thermo Scientific, Trace GC Ultra/ISQ interfaced with a Single Quadrupole MS, TG-5MS fused silica capillary column (30 m, 0.251 mm, 0.1 mm film thickness) as follows: an electron ionisation system was run in electron impact mode with ionisation energy of 70 eV. Helium gas (99.999%) was used as the mobile phase at a constant flow rate of 1 mL/min, and an injection volume of 2 μL was utilised (a split ratio of 10:1). The injector temperature and the ion-source temperature were maintained at 250 °C. In the GC part, the oven temperature was set up at 110 °C (isothermal for 2 min), with an increase of 10 °C/min until reaching 200 °C, followed by a 5 °C/min increase to score 280 °C, at which the oven temperature was kept isothermal for 9 min. Mass spectra were taken at 70 eV; a scan interval of 0.5 s and fragments from 45 to 450 Da. The solvent delay was 0–2 min, and the total GC-MS running time was 36 min. The relative percentage amount of each component was calculated by comparing its average peak area to the total area. Processing of data was carried out using GC-MS solution software. The compounds were identified by comparing their mass spectra to Wiley and the National Institute of Standards and Technology (NIST) Libraries (Adams 2007).

### Molecular docking against thymidylate kinase (TMK)

The major identified compounds, 2-naphthonitrile, 5,6,7,8-tetrahydro (**1**), 2-benzenediacetonitrile (**2**), quinoline, 2-ethenyl (**3**), 2-[1-(4-cyano-1,2,3,4-tetrahydronaphthyl)] propanenitrile (**4**), octadecane, 1,1-dimethoxy (**5**), 9,12-octadecadienoic acid (*Z*,*Z*), methyl ester (**6**), and benzene, 1,1′-(1,3-butadiene-1,4-diyl)*bis* (**7**), from the *Ts*-EtOAc GC-MS analysis, were docked against the active sites of the thymidylate kinase enzyme (TMK) from *S. aureus* using the Molecular Operating Environment (MOE; Chemical Computing Group ULC, Montreal, QC, Canada). The co-crystal structure of TMK was obtained from the Protein Data Bank (PDB ID 4QGG; www.rcsb.org/pdb). For validation, redocking of the co-crystallized ligand was first carried out, displaying a docking score value of −9.632 kcal/mol with a Root Mean Square Deviation (RMSD) of 1.7 Å. The chemical structures of identified compounds were built and saved in their 2 D and 3 D conformations by the Builder tool incorporated into MOE 2014.0901. Then, the energy of the docked structures was minimised using the MMF94FX force field with a gradient Root Mean Square (RMS) of 0.0001 kcal/mol. A docking simulation of examined compounds in the active binding site of TMK was then performed. Water molecules were deleted during the docking process, and the missing hydrogen atoms were retained to correct ionisation states to be assigned to the protein structure. The ‘Docking’ module in MOE was run to achieve molecular docking. The docking process was applied with the default settings. The top 30 poses, as ranked by London dG, were saved, and then GBVI/WSA dG (Generalized-Born Volume Integral/Weighted Surface Area) scoring function was applied to score the resultant poses. The ‘Ligand Interactions’ MOE tool for analysis of docking results was utilised by visualisation of the protein-ligand interactions inside the active site (Barakat et al. 2018).

### Statistical analysis

The data are presented as mean ± standard deviation (SD). One-way analysis of variance (ANOVA) test was used for statistical analyses in SPSS v.20.0 (Statistical Program for Social Sciences software; SPSS Inc. USA). The significance of the data was determined using Duncan’s multiple range test. *p* Values less than 0.05 were considered statistically significant.

## Results

### Total antioxidant capacity, total phenolics and free radical scavenging activity

Screening of different solvent extracts of *T. savignyi* for their antioxidant potential showed that ethyl acetate extract (*Ts*-EtOAc) was the highest in total antioxidant capacity, followed by acetone extract, with 551.33 and 532.0 mg AAE/g dry weight of extract, respectively. However, the methanol extract was the lowest in total antioxidant capacity compared to the reference antioxidant (ascorbic acid), acetone, ethanol, and *Ts*-EtOAc extracts. The same order of antioxidant powerfulness was also observed when comparing the total phenolic contents (TPC) of each extract. For example, *Ts*-EtOAc gave the highest yield of total phenolics with a TPC value of 254.46 mg GAE/g dry weight of extract, and methanol extract had the lowest TPC (150.28 mg GAE/g). Moreover, the free radical scavenging activity, measured by the DPPH assay, of *Ts*-EtOAc extract was the highest, with a 50% inhibition (IC_50_) at 24.0 µg/mL concentration. Overall, *Ts*-EtOAc extract has the most potent antioxidant activity among all the extracts tested (Table 1).

**Table 1.** Total antioxidant capacity (TAC) and total phenolic content (TPC), and free radical scavenging activity (DPPH) values of different solvent extracts of *Thais savignyi* snails.

Extract	TAC (mg AAE/g)^a^	TPC (mg GAE/g)^b^	DPPH (IC_50_ µg/mL)^c^
*Ts*-MeOH	482.72 ± 7.74	150.28 ± 3.45	32.36 ± 2.37
*Ts*-Acetone	532.0 ± 2.0	209.51 ± 4.75	37.52 ± 1.68
*Ts*-EtOAc	551.33 ± 3.05	254.46 ± 5.63	24.0 ± 1.25
*Ts*-EtOH	492.66 ± 5.03	203.24 ± 3.44	46.92 ± 2.18
Ascorbic acid			7.50 ± 1.50

^a^AAE (ascorbic acid equivalent); ^b^GAE (gallic acid equivalent); ^c^IC_50_: The amount of extract needed to scavenge 50% of DPPH radicals; *Ts*: *Thais savignyi*

### Antimicrobial activity

The *in vitro* antimicrobial activity of the crude solvent extracts of *T. savignyi* was tested against several human pathogenic microorganisms, including five Gram-negative bacteria, one Gram-positive bacterium, and one pathogenic fungal strain. The results showed that, among the tested extracts, only *Ts*-EtOAc exhibited broad antimicrobial activity with strong antibacterial activity against *K. pneumoniae* with antibacterial activity up to 87.65% inhibition, and moderate antibacterial activity towards each of *P. vulgaris*, *P. aeruginosa,* and *S. aureus* with inhibition activities of 64.94, 59.44, and 67.19%, respectively. Concerning the extract activity against the fungal strain tested, it showed a pronounced antifungal activity against *C. albicans* with an inhibition percentage of 84.02%. The other tested extracts showed low or no activity against the tested microbes (Table 2). Additionally, the MIC of the *Ts*-EtOAc extract was also evaluated and presented in Table 3. *Ts*-EtOAc demonstrated the highest activity against *K. pneumoniae* and *C. albicans* with MIC of 3.90 μg/mL.

**Table 2.** Antibacterial inhibition (%) of *Thais savignyi* snail extracts against various bacteria and fungi.

Extract	*P. vulgaris*	*E. coli*	*P. aeruginosa*	*C. albicans*	*K. pneumoniae*	*S. aureus*
*Ts*-MeOH	10.78 ± 0.07	30.18 ± 0.25	17.30 ± 0.12	0.00	0.00	46.76 ± 0.27
*Ts*-Acetone	0.00	0.00	0.00	0.00	0.00	0.00
*Ts*-EtOAc	64.94 ± 1.89	44.54 ± 0.90	59.44 ± 5.96	84.02 ± 0.44	87.65 ± 0.46	67.19 ± 0.46
*Ts*-EtOH	0.00	0.000	0.00	0.000	0.000	4.264 ± 0.51

**Table 3.** Minimum inhibitory concentration (MIC) of *Ts*-EtOAc extract and a standard drug against various bacterial pathogens.

Bacterial pathogens	Minimum inhibitory concentration (MIC) in μg/mL	
	*Ts*-EtOAc	Ciprofloxacin
*Proteus vulgaris*	15.62	0.780
*Escherichia coli*	15.62	0.390
*Pseudomonas aeruginosa*	15.62	3.12
*Klebsiella pneumoniae*	3.90	0.390
*Staphylococcus aureus*	7.81	6.24
*Candida albicans*	3.90	–

**Figure 1.**
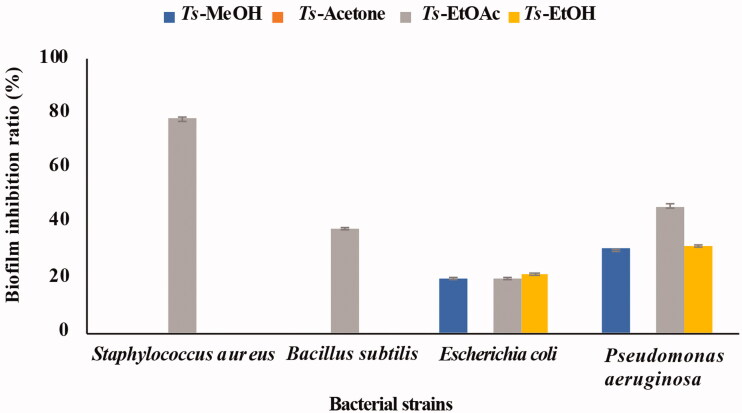
Biofilm inhibitory activities of different solvent extracts of *Thais savignyi* snails.

The ability of the *T. savignyi* organic extracts to eradicate bacterial biofilm formation by four pathogenic bacteria (*S. aureus*, *P. aeruginosa*, *B. subtilis,* and *E. coli*) was measured using the MTT assay. *Ts*-EtOAc inhibited biofilm formation by *S. aureus* with an inhibition ratio close to 80% and showed moderate biofilm inhibition against *B. subtilis* and *P. aeruginosa*. However, it showed low biofilm inhibition activity towards *E. coli*. *Ts*-EtOH extract showed only low biofilm inhibition activity against *E. coli* and *P. aeruginosa* (Figure 1).

### Cytotoxicity

**Figure 2.**
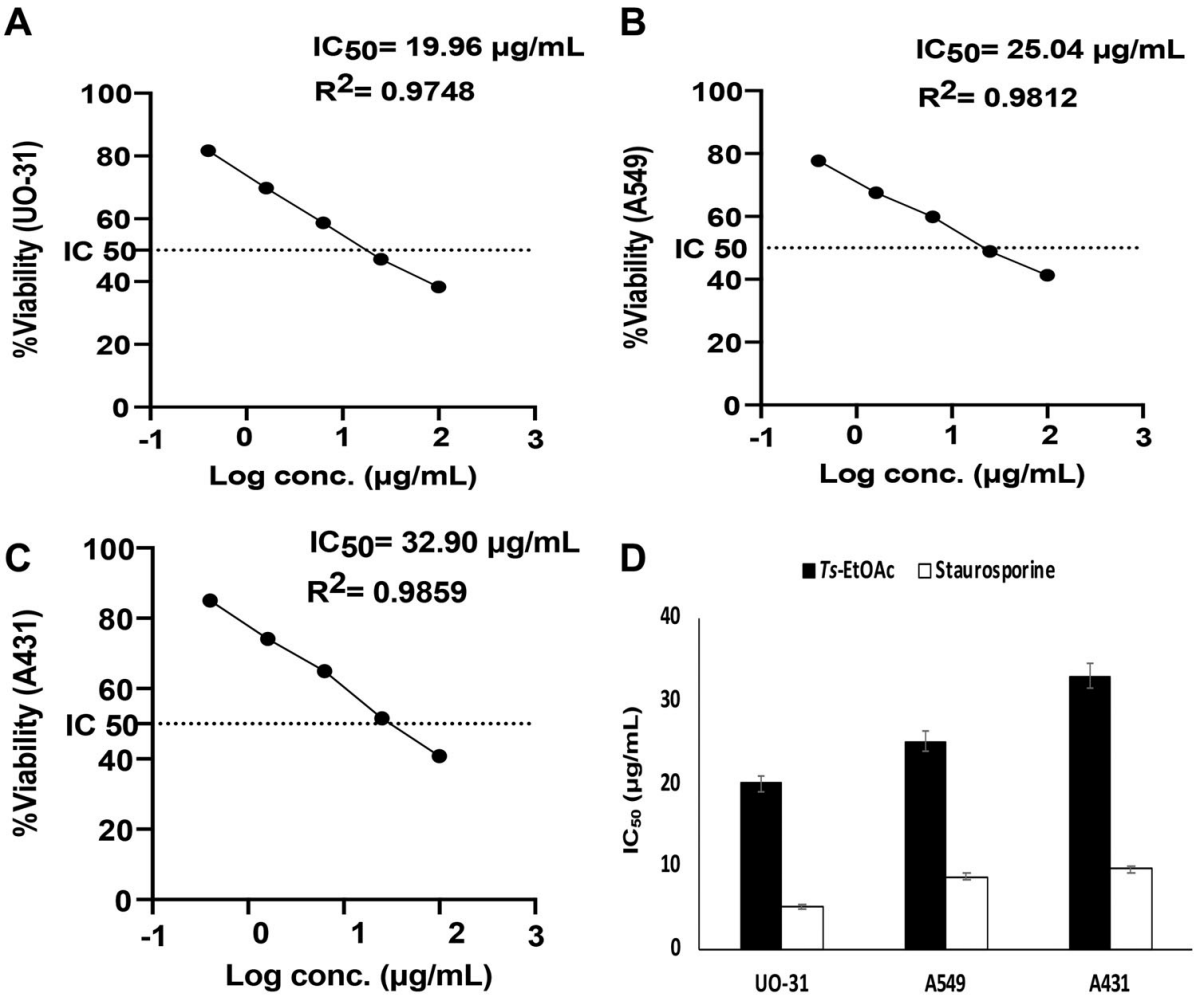
Cytotoxicity of *Ts*-EtOAc extract against different human cancer cell lines after 24 h of treatment. **(A)** UO-31: Human kidney renal cell carcinoma; **(B)** A549: Adenocarcinomic human alveolar basal epithelial cells; **(C)** A431: Human epidermoid carcinoma cells (*X*-axis: Log concentrations of the extract from 0.4 to 100 μg/mL and *Y*-axis: the percentage of cell viability). **(D)**: The comparison of average IC_50_ of the extract vs. staurosporine as a positive control. Data represent the mean ± SD of three independent experiments.

The *in vitro* cytotoxic effects of *Ts*-EtOAc extract against human kidney renal cell carcinoma UO-31, adenocarcinomic human alveolar basal epithelial cells A549 and human epidermoid carcinoma cells A431 were examined using the MTT assay and compared to control. The *Ts*-EtOAc extract showed variable toxicity towards UO-31, A549, and A431 cell lines. A dose dependent decrease in cell viability was observed (Figure 2). The highest observed effect was against UO-31 with an IC_50_ value of 19.96 ± 0.93 μg/mL compared to 5.165 ± 0.24 μg/mL for the control drug (Figure 2A,D). The lowest effect was observed against the A431 cell line. Figure (2D) shows the IC_50_ values for *Ts*-EtOAc extract vs. staurosporine as a positive control against cancer cell lines investigated. Overall, the extract showed moderate toxicity against all cell lines examined (Figure 2).

### GC-MS investigation of Ts-EtOAc extract

**Figure 3.**
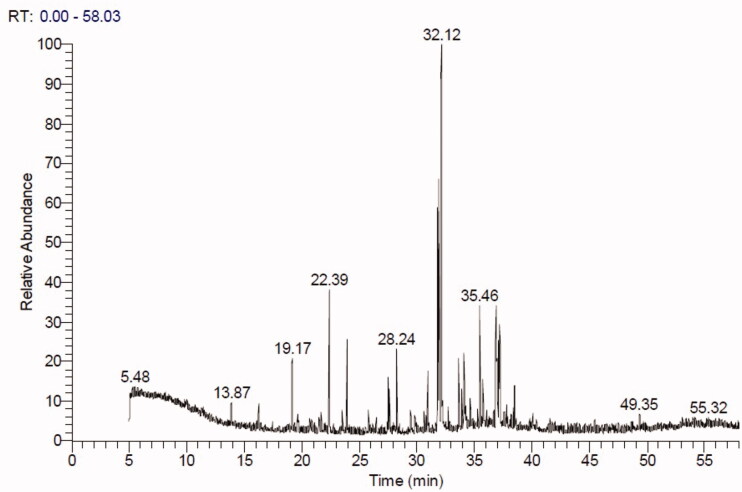
GC-MS chromatogram of *Ts*-EtOAc extract.

**Figure 4.**
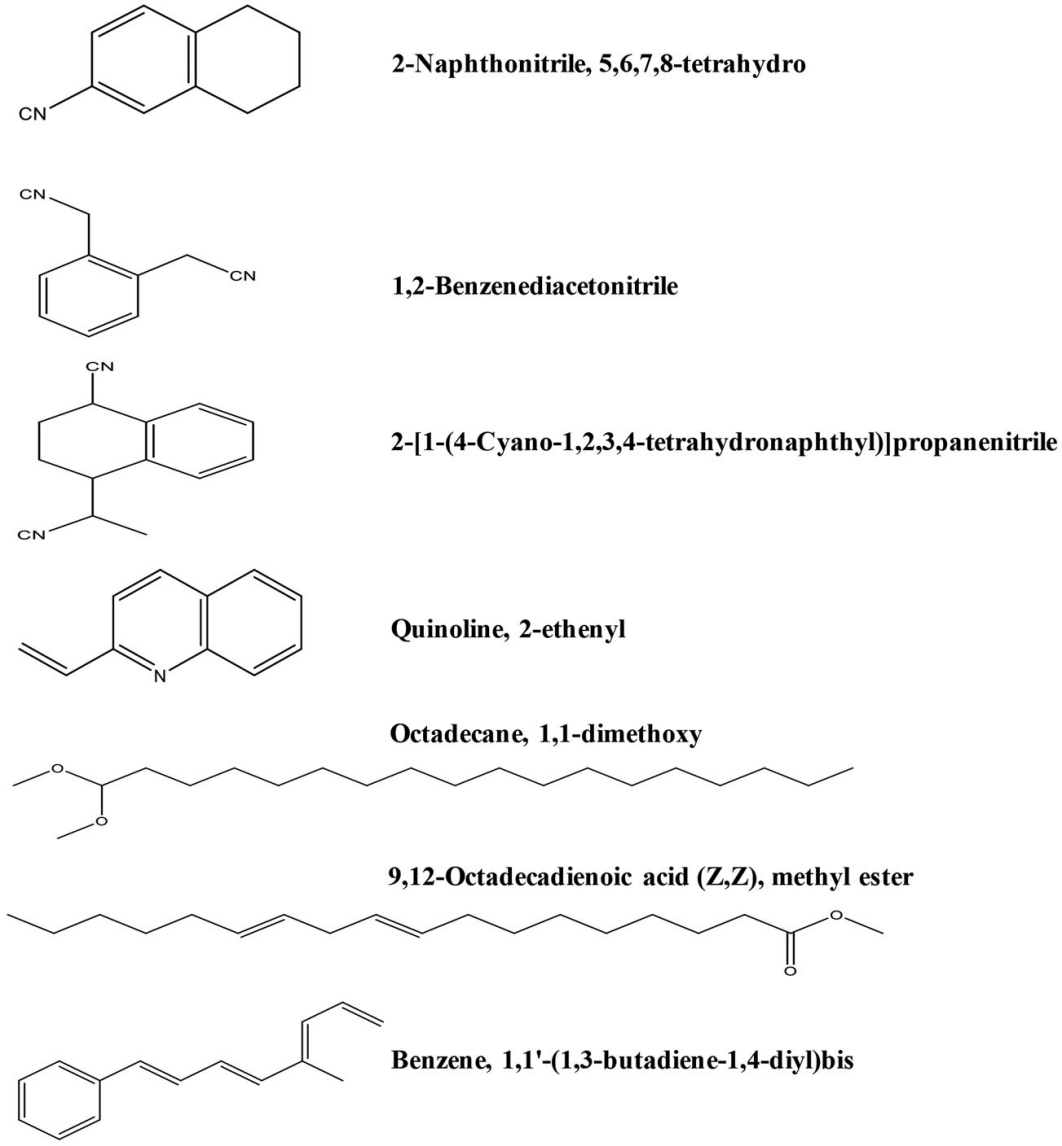
Chemical structures of some major identified compounds in *Ts*-EtOAc extract.

The GC-MS chromatogram of ethyl acetate extract of *T. savignyi* (*Ts*-EtOAc) is shown in Figure 3. GC-MS analysis of *Ts*-EtOAc extract identified 45 compounds. The total peak areas of the identified ingredients constitute 95.82% of the total extract composition. The proposed chemical structures of the identified compounds are recorded in Table 4. The chemical composition of *Ts*-EtOAc extract is characterised by a high representation of 2-naphthonitrile, 5,6,7,8-tetrahydro with 17.19% followed by 1,2-benzenediacetonitrile (8.67%) and 2-[1-(4-cyano-1,2,3,4-tetrahydronaphthyl)] propanenitrile (8.38%). Other major compounds identified include quinoline, 2-ethenyl (6.39%), (1a′,4a′,4aa′,10aa′)-1,4,4a,5,6,7,8,9,10,10a-decahydro-1,4,11,11-tetramethyl-1,4 methanocycloocta[d]pyridazine (4.0%), octadecane, 1,1-dimethoxy (3.79%), 9,12-octadecadienoic acid (*Z*,*Z*), methyl ester (3.60%), and benzene, 1,1′-(1,3-butadiene-1,4-diyl)*bis* (2.79%). The aforementioned compounds collectively represented 54.81% of the overall peak areas (Figure 4).

**Table 4.** Chemical composition of *Ts*-EtOAc extract.

No.	Rt	Area%	M.W.	M.F.	Main Fragments	Identified compounds	Class/category
**1**	32.12	17.19	157	C_11_H_11_N	63, 88, 115, 129, 154	2-Naphthonitrile, 5,6,7,8-tetrahydro	Naphthalene derivatives
**2**	31.79	8.67	156	C_10_H_8_N_2_	77, 102, 129, 156	1,2-Benzenediacetonitrile	Nitriles
**3**	36.92	6.39	155	C_11_H_9_N	129, 154, 155	Quinoline, 2-ethenyl	Quinoline derivatives
**4**	31.9	8.38	210	C_14_H_14_N_2_	51, 63, 77, 102, 129, 156, 180	2-[1-(4-Cyano-1,2,3,4-tetrahydronaphthyl)] propanenitrile	Naphthalene derivatives
**5**	35.46	3.79	314	C_20_H_42_O_2_	71, 75, 95, 111, 250, 283	Octadecane, 1,1-dimethoxy	Alkane derivatives
**6**	34.08	3.6	294	C_19_H_34_O_2_	41, 55, 67, 81, 95, 110, 123, 263	9,12-Octadecadienoic acid (*Z*,*Z*), methyl ester	Fatty acid esters
**7**	37.21	2.79	206	C_16_H_14_	77, 91, 115, 129, 152, 178, 191	Benzene, 1,1′-(1,3-butadiene-1,4-diyl)*bis*	Stilbenoids
**8**	5.09	1.51	188	C_10_H_20_OS	57, 69, 84, 110, 146	Nonanal, 3-(methylthio)	Alpha-hydrogen aldehydes
**9**	13.87	0.78	182	C_13_H_26_	41, 43, 55, 69, 83, 168	1-Tridecene	Acyclic olefin
**10**	16.25	0.89	198	C_14_H_30_	41, 43, 57, 71, 85, 113, 155, 169	Decane, 2,3,5,8-tetramethyl	Branched alkanes
**11**	19.17	2.12	200	C_13_H_28_O	55, 69, 83, 97, 111, 125, 154	1-Tridecanol	Long-chain fatty alcohols
**12**	19.67	0.82	170	C_11_H_22_O	29, 41, 57, 67, 82, 133, 152	*trans*-2-Undecen-1-ol	Fatty alcohols
**13**	20.7	0.8	192	C_12_H_16_O_2_	29, 51, 57, 77, 86, 105, 145, 175	3-Pentanone, 1-hydroxy-2-methyl-1-phenyl	Phenylpropanes
**14**	21.48	0.78	194	C_10_H_10_O_4_	51, 77, 104, 118, 131, 148, 175	Butanedioic acid, phenyl	Phenylpropanoic acids
**15**	21.7	0.55	198	C_14_H_30_	43, 57, 71, 85, 99, 113, 127, 169	Tetradecane	Straight chain alkanes
**16**	22.39	4	234	C_15_H_26_N_2_	57, 91, 115, 191, 206	(1a′,4a′,4aa′,10aa′)-1,4,4a,5,6,7,8, 9,10,10a-decahydro-1, 4,11,11-tetramethyl-1,4 methanocycloocta [d]pyridazine	Pyridazine derivatives
**17**	23.5	0.89	168	C_12_H_24_	41, 43, 55, 70, 83, 91, 139	5-Undecene, 3-methyl, (Z)	Unsaturated aliphatic hydrocarbons
**18**	23.94	2.59	214	C_14_H_30_O	55, 57, 69, 83, 97, 111, 125	1-Tetradecanol	Long-chain fatty alcohols
**19**	25.79	0.77	154	C_11_H_22_	55, 70, 83, 97, 112, 128, 149	5-Ethyl-1-nonene	Branched alkenes
**20**	26.15	0.43	320	C_21_H_36_O_2_	43, 67, 79, 107, 150, 205, 222,289	8,11,14-Eicosatrienoic acid, methyl ester	Fatty acid methyl esters
**21**	26.51	0.41	268	C_19_H_40_	43, 57, 71, 85, 99, 113, 141, 197	Nonadecane	Straight-chain alkanes
**22**	27.5	1.9	238	C_15_H_26_O_2_	41, 43, 71, 93, 107,121, 143, 169, 184, 220	Bisabolol oxide A	Sesquiterpenoids
**23**	27.59	1.7	208	C_16_H_16_	51, 65, 89, 104, 128, 177	Benzene,1,1′-(1,2-cyclobutanediyl)bis-, trans	Stilbenoids
**24**	28.24	2.4	252	C_18_H_36_	55, 69, 97, 111, 125, 139, 231	5-Octadecene, (*E*)	Unsaturated aliphatic hydrocarbons
**25**	29.45	0.88	174	C_11_H_10_O_2_	58, 91, 115, 145	4-(2-Methylphenyl)furan-2(5H)-one	Toluenes
**26**	29.83	0.87	118	C_9_H_10_	91, 103, 115, 117	Pentacyclo[6.1.0.0(1, 7).0(2,8).03, 5)]nonane	Alkanes
**27**	30.61	0.61	158	C_12_H_14_	39, 51, 77, 104, 115, 129	Benzene, 3-cyclohexen-1-yl	Benzene and substituted derivatives
**28**	30.82	0.47	310	C_22_H_46_	43, 57, 71, 85, 113, 127, 169, 197, 253	Docosane	Straight-chain alkanes
**29**	30.96	1.98	270	C_17_H_34_O_2_	29, 41, 43, 74, 87, 143, 185, 227	Hexadecanoic acid, methyl ester	Fatty acid esters
**30**	32.71	0.54	240	C_16_H_32_O	41, 55, 71, 82, 97, 111, 152, 222	Oxirane, tetradecyl	Epoxides
**31**	33.61	2.43	144	C_11_H_12_	51, 63, 77, 102, 115, 129	Cyclopropene, 2,3-dimethyl-3-phenyl	Cycloalkene derivatives
**32**	33.89	1.32	210	C_15_H_30_	41, 43, 55, 70, 83, 97, 111, 182, 210	Pentadecene	Unbranched alkene
**33**	34.19	1.07	296	C_19_H_36_O_2_	55, 67, 83, 97, 125, 180, 264	9-Octadecenoic acid, methyl ester	Fatty acid esters
**34**	34.64	0.78	298	C_19_H_38_O_2_	43, 55, 74, 87, 129, 143, 199, 255	Octadecanoic acid, methyl ester	Fatty acid esters
**35**	35.73	1.85	242	C_16_H_34_O	29, 41, 43, 55, 69, 83, 97, 111, 125, 196	1-Hexadecanol	Fatty alcohol
**36**	36.07	0.43	256	C_17_H_36_O	43, 57, 69, 71, 83, 111, 210, 238	1-Hexadecanol, 2-methyl	Fatty alcohol derivatives
**37**	36.69	0.58	154	C_12_H_10_	102, 127, 151, 153	Benzene, (2,4-cyclopentadien-1 -ylidenemethyl)	Benzene and substituted derivatives
**38**	36.99	2.09	156	C_9_H_4_N_2_O	128, 155	2,6-Dicyanobenzaldehyde	Aldehyde derivatives
**39**	37.13	2.49	205	C_15_H_11_N	165, 178, 190, 204	9-Phenanthrenecarbonitrile, 9,10-dihydro	Phenanthrene derivatives
**40**	37.59	0.47	362	C_26_H_50_	43, 67, 82, 95, 137, 181, 292, 305	1,1′-Bicyclopentyl, 2-hexadecyl	Iridoid derivatives
**41**	37.8	0.64	314	C_19_H_38_O_3_	29, 55, 85, 97, 113, 180, 220, 264	Octadecanoic acid, 6-hydroxy, methyl ester	Fatty acid esters
**42**	38.22	0.46	154	C_10_H_18_O	29, 41, 55, 67, 84, 110, 136, 147	*cis*-4-Decenal	Long-chain aldehydes
**43**	47.06	0.8	446	C_28_H_46_O_4_	27, 41, 55, 69, 83, 97, 139	3-Isobutyl-1- Methylcyclopentanone	Cyclic ketones derivatives
**44**	40.07	0.4	194	C_12_H_18_O_2_	31, 41, 55, 77, 91, 115, 124, 163	5,7-Dodecadiyn-1,12-diol	Alkyne derivatives
**45**	45.46	0.51	136	C_10_H_16_	41, 53, 68, 79, 93, 107, 121	Limonene	Cyclic monoterpenes
95.82%							

Rt: Retention time; M.W.: molecular weight; M.F.: molecular formula.

### Molecular docking analysis

**Figure 5.**
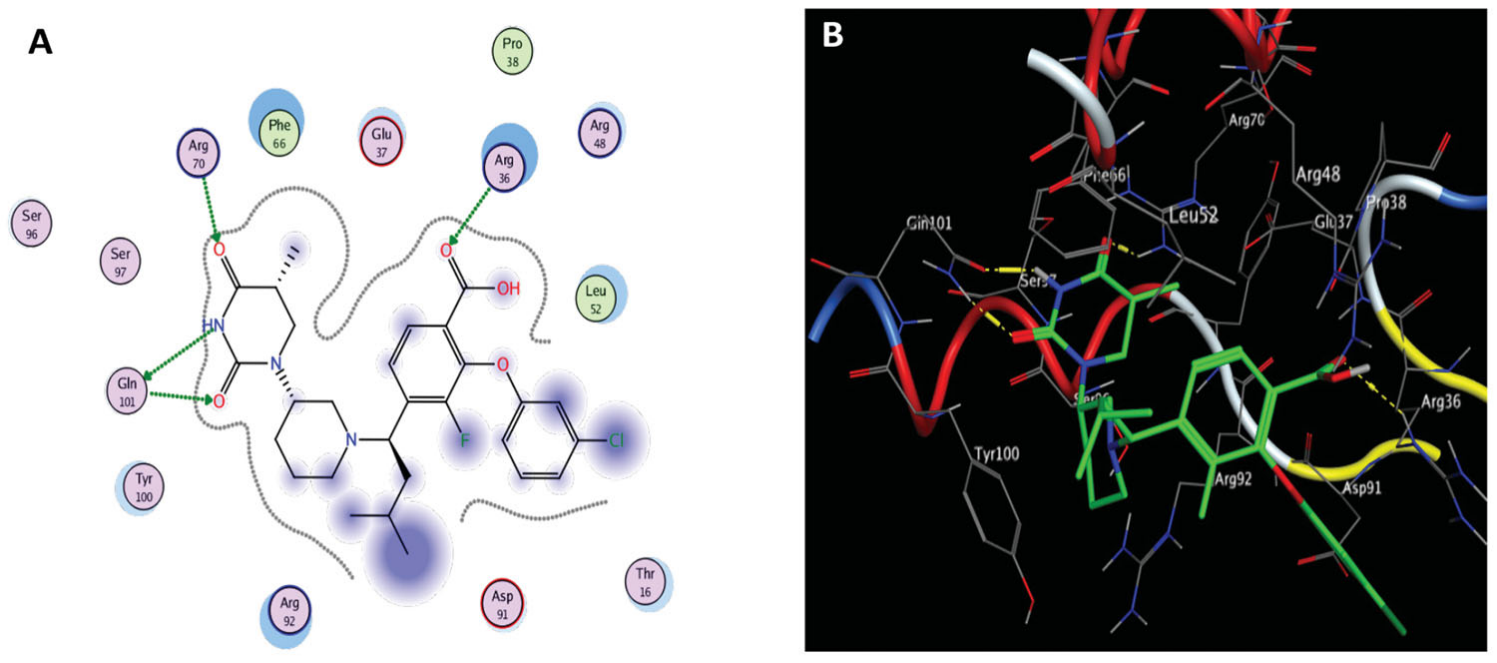
The two-dimensional (A) and three-dimensional (B) suggested binding modes of redocked ligand within the binding pocket of TMK (PDB: 4QGG).

**Figure 6.**
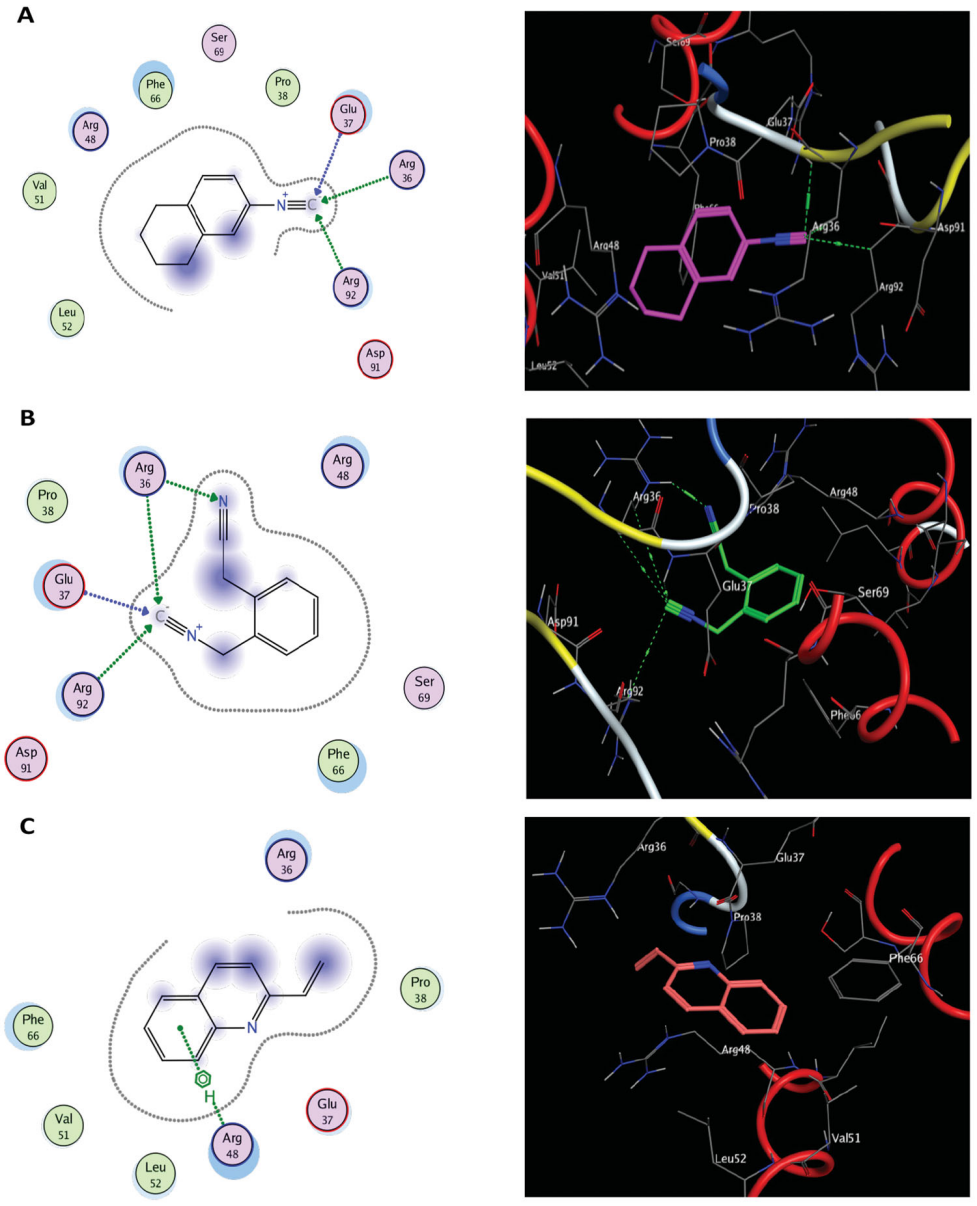
The two-dimensional (left panel) and three-dimensional (right panel) suggested binding modes of compounds **1** (A), **2** (B) and **3** (C) within the binding pocket of TMK (PDB: 4QGG).

**Figure 7.**
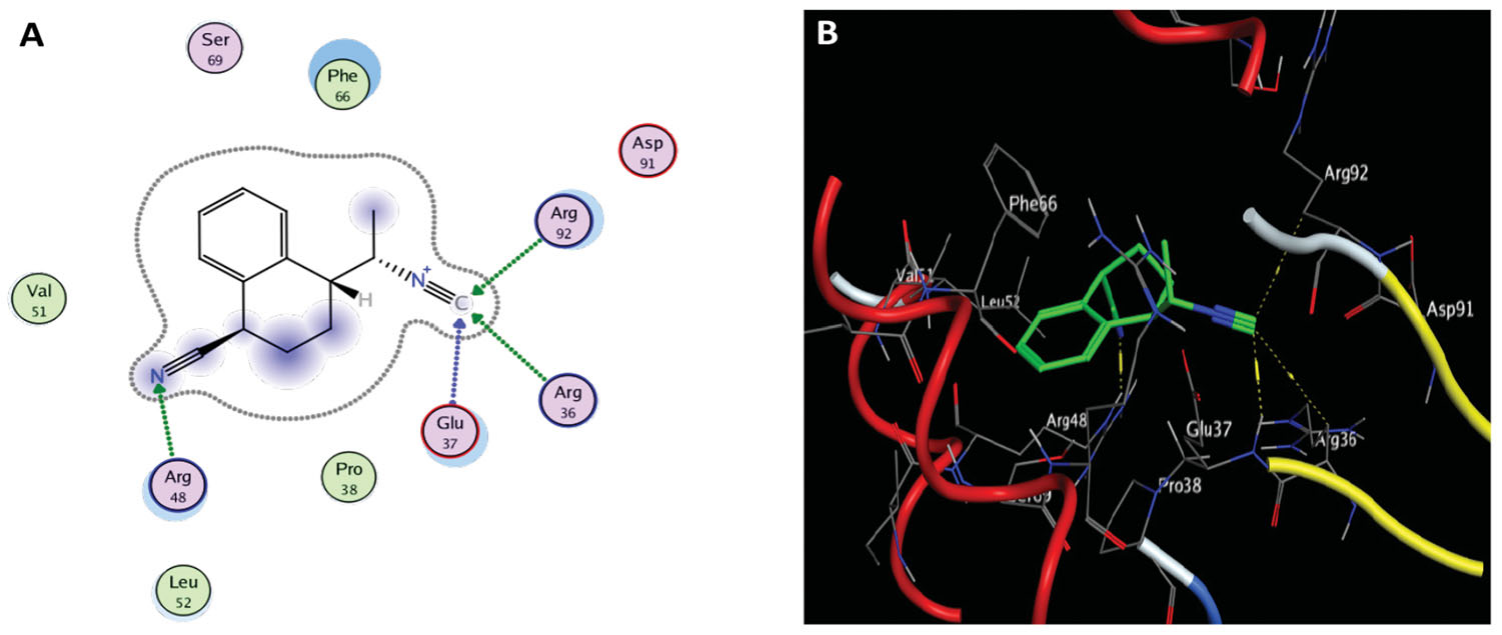
The two-dimensional (A) and three-dimensional (B) suggested binding modes of compound **4** within the binding pocket of TMK (PDB: 4QGG).

**Figure 8.**
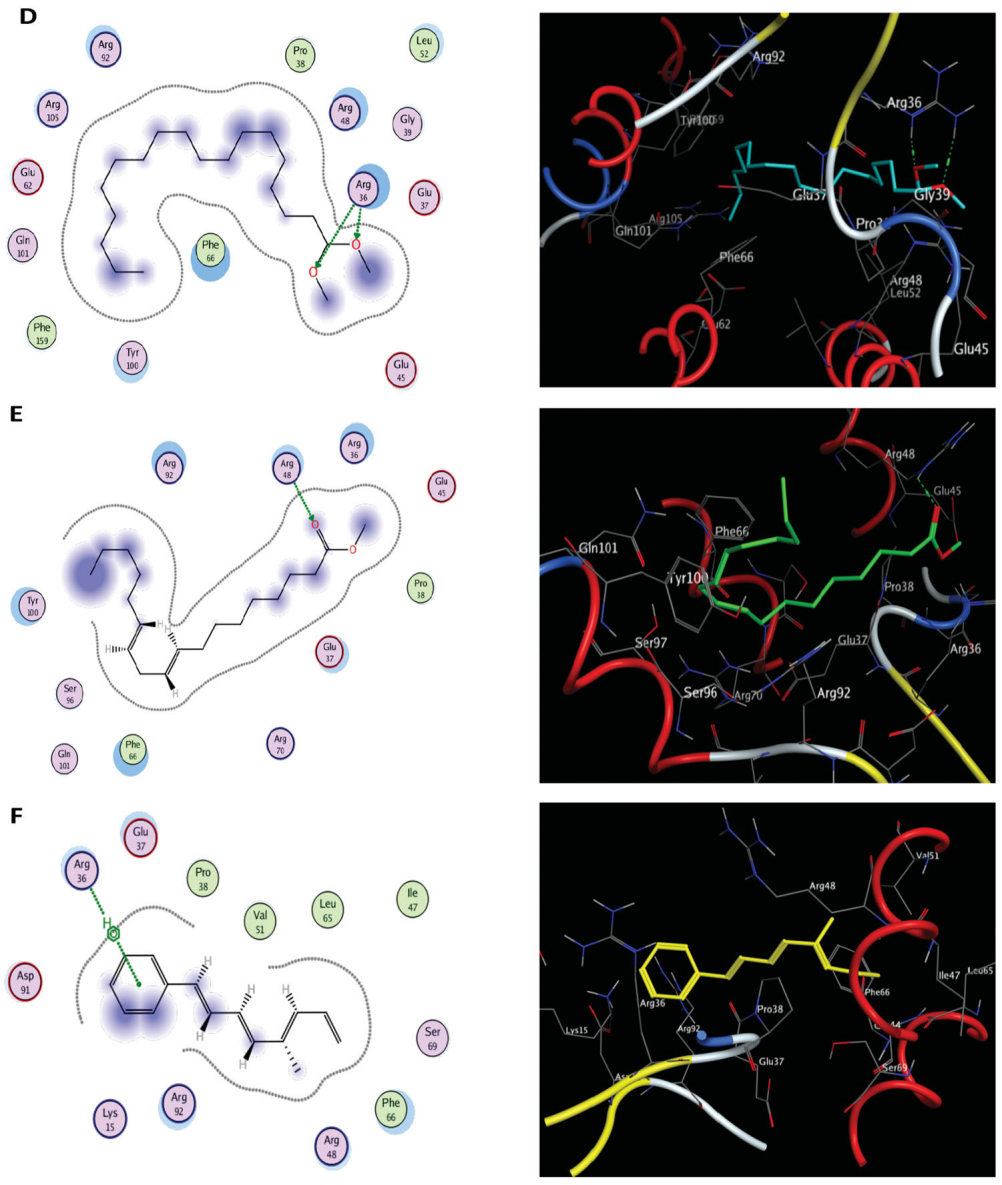
The two-dimensional (left panel) and three-dimensional (right panel) suggested binding modes of compounds **5** (D), **6** (E) and **7** (F) within the binding pocket of TMK (PDB: 4QGG).

Molecular docking results of spectrometrically identified compounds (**1-7**) inside the active site of thymidylate kinase (PDB: 4QGG) are represented in Table 5 and Figures 5–8. The anticipated active site residues of the target protein were Arg36, Arg48, Arg70, Arg92, Gln37, and Gln101 amino acids. Redocking of the co-crystallized ligand yielded a docking score value of −9.632 kcal/mol with a RMSD of 1.7 Å. Four hydrogen bonds were implicated in the interaction of the ligand with TMK residues; one bond with each of Arg36 and Arg70 and two bonds with the crucial residue Gln101 (Figure 5A,B). Compounds under examination demonstrated binding energies scores ranging from −3.004 to −9.117 kcal/mol. Compounds **1** (Figure 6A), **2** (Figure 6B), **4** (Figure 7A,B), **5** (Figure 8D), **6** (Figure 8E) demonstrated good binding interactions with the most critical residues in the active site, as predicted by the docking scores and their binding poses. Compound **4** (2-[1-(4-cyano-1,2,3,4-tetrahydronaphthyl) propanenitrile) with the best binding energy score, recording −9.117 kcal/mol, demonstrated strong binding interaction inside the active binding region via the creation of four hydrogen binds with Arg36, Arg48, Arg92, and Gln37 (Figure 7A,B).

**Table 5.** The docking scores (kcal/mol) and binding interactions of compounds (1**–**7) from *Ts*-EtOAc extract within the active binding sites of TMK (PDB: 4QGG).

N	Compound	Docking score (kcal/mol)	Binding interactions	
			Residue	Type
**1**	2-Naphthonitrile, 5,6,7,8-tetrahydro	−7.223	Arg36	3 hydrogen bonds
			Arg92	
			Gln37	
**2**	1,2-Benzenediacetonitrile	−8.184	Arg36	4 hydrogen bonds
			Arg92	
			Gln37	
**3**	Quinoline, 2-ethenyl	−3.233	Arg48	Arene-H
**4**	2-[1-(4-Cyano-1,2,3,4-tetrahydronaphthyl)] propanenitrile	−9.117	Arg36	4 hydrogen bonds
			Arg48	
			Arg92	
			Gln37	
**5**	Octadecane, 1,1-dimethoxy	−6.788	Arg36	2 hydrogen bonds
**6**	9,12-Octadecadienoic acid (*Z*,*Z*), methyl ester	−6.249	Arg48	1 hydrogen bond
**7**	Benzene, 1,1′-(1,3-butadiene-1,4-diyl)*bis*	−3.004	Arg36	Arene-H
**Ligand**		−9.632	Arg36	4 hydrogen bonds
			Arg70	
			Gln101	

## Discussion

Marine organisms are rich in structurally diverse bioactive materials with drug development potential. Bioactive substances isolated from marine fauna have antiviral, antimicrobial, antiprotozoal, antifungal, anthelmintic, and anticancer activities (Pangestuti and Kim 2017). Secondary metabolites derived from marine molluscs have a wide range of pharmaceutical applications (Benkendorff 2010; Odeleye et al. 2019). In the present investigation, the gastropod snail, *Thais savignyi*, collected from the Red Sea, was extracted with different organic solvents and its antioxidant, antimicrobial, and cytotoxic activities were evaluated.

### Antioxidant activity of Ts-EtOAc extract

Reactive oxygen species (ROS) are implicated in many disorders and ailments (Brieger et al. 2012). Maintaining the homeostasis of ROS production and antioxidants within the cells is crucial for disease prevention. Complementary antioxidants (dietary components or medications) are recommended to overcome the antioxidant deficiency. In this regard, medicinal plants and marine organisms have been extensively studied for their antioxidant power (Xu et al. 2017; Chakraborty and Joy 2020). In the present investigation, the tissue extracts of *T. savignyi* were compared for their antioxidant capacity. The results showed that *Ts*-EtOAc was the best extract in total antioxidant capacity and DPPH free radical scavenging activities. Although EtOAc is a semi-polar solvent, it yielded the highest phenolic content among the other extracts tested, including methanol. This may be due to the specific nature of *T. savignyi* tissues. The IC_50_ for *Ts*-EtOAc was 24.0 µg/mL. The smaller the IC_50_ value (concentration required to inhibit 50% of DPPH free radicals), the higher the free radical scavenging activity of the extract (Molyneux 2004). Other mollusc extracts showed lower antioxidant activities compared to *Ts*-EtOAc. For example, the IC_50_ value for the methanol extract of the marine snail, *Pleuroploca trapezium* L. (Fasciolariidae), was 4021 μg/mL (Prem Anand et al. 2010). Moreover, crude peptide extract of the *Patella rustica* L. (Patellidae) snail showed a scavenging ability of 79.77% at 0.39 mg/mL against the DPPH radical (Borquaye et al. 2015). The antioxidant activities (expressed as IC_50_) of the methanolic extract of two marine bivalves, *Perna viridis* L. (Mytilidae) and *Meretrix meretrix* L. (Veneridae), were 247.85 µg/mL and 84.46 µg/mL, respectively (Krishnamoorthy et al. 2019; Minsas et al. 2020).

Many bioactive secondary metabolites bearing phenolic moieties have been identified in marine molluscs (Salas and Chakraborty 2020). Phenolic compounds possess potent antioxidant and radical scavenging characteristics and can pre-empt the progress of many diseases (Dias et al. 2021). Therefore, the antioxidant potency of *Ts*-EtOAc compared to other solvent extracts investigated may be due to its high content of phenolics (254.46 mg/g). Similarly, ethyl acetate-methanol extracts of the muricid gastropod *Chicoreus ramosus* L. and *Babylonia spirata* L. (Babyloniidae) had higher phenolic content than chloroform extract of the same species. Moreover, the high phenolic content of ethyl acetate-methanol was associated with antioxidant preferences in scavenging DPPH radicals (Salas and Chakraborty 2020).

### Antimicrobial activity

The emergence and propagation of antimicrobial resistance are today’s most significant health concerns. The marine environment is a rich source of new compounds critical for the treatment of drug-resistant bacterial diseases (Liu et al. 2019). Molluscs are largely benthic organisms that live clinging to surfaces, making them vulnerable to microbial and viral invasions. Due to the absence of an acquired immune system, these animals must have developed alternate defense mechanisms against the assault of microbial invasions. Molluscs may have evolved a variety of antibacterial, antifungal, antiparasitic, and antiviral secondary metabolites for circulation in the haemolymph and inclusion in mucus secretions on the body surface (Benkendorff 2010). As a result, molluscs are a rich source of antibacterial chemicals (Benkendorff et al. 2004). The results of antibacterial screenings of different extracts of *T. savignyi* indicated that *Ts*-EtOAc was the most powerful against all tested microbes, with MIC values ranging from 15.62 μg/mL in the case of *P. aeruginosa* up to 3.90 μg/mL against *K. pneumoniae*. Methanol, ethanol, and acetone extracts were of either low or negative antibacterial activity against the tested pathogens. Kumaran et al. (2011) discovered that the ethyl acetate extract of *Thais tissoti* Petit de la Saussaye (Muricidae) was more effective against *K. pneumoniae* and *S. aureus* than the methanol extract. Similarly, ethyl acetate of the marine snail, *B. spirata,* was more effective than methanol or acetone extracts against *E. coli*, *K. pneumoniae*, and *S. aureus* (Kumaran et al. 2011). Likewise, Prem Anand and Edward (2002) found that the ethyl aetate extract of the marine gastropod species *Cypraea errones* L. (Cypraeidae) had antimicrobial activity against *P. aeruginosa* and *S. aureus* compared to a heptane extract that had no activity against the same microbes.

The present results also showed that *Ts*-EtOAc was active against the fungus *C. albicans* with MIC value of 3.90 μg/mL. Crude peptide extract of *P. rustica* was also effective in inhibiting the growth of *C. albicans* at concentrations comparable to the positive control (37 mm for *P. rustica* against 40 for the standard drug ciprofloxacin) (Borquaye et al. 2015).

The advantage of EtOAc extract over methanol extract could be attributed to the former’s lower polarity, which allows for the presence of fatty acids and lipids in the extract due to their lipophilic nature. According to Karthikeyan et al. (2014), ethyl acetate extract of the oyster *Saccostrea glomerata* Gould (Ostreidae) is a high source of fatty acids, which could explain its antibacterial potential. However, microbial responses to diverse solvent extracts are mediated by a unique interaction with specific chemicals in the studied extract, which could explain why one solvent extract can inhibit some microorganisms while others cannot. Because the types and amounts of bioactive chemicals differ from one species to another, variances in antibacterial potentials between mollusc species extracted with comparable or variable solvents are expected. Unlike the present finding that EtOAc is the best solvent for the extraction of *T. savignyi*, producing significant antibiotic activity, the antibacterial activities of ethanol extracts of other species, such as the gastropods *B. spirata* and *Turbo brunneus* Röding (Turbinidae), were the strongest against *E. coli*, *K. pneumoniae*, *P. vulgaris*, and *Salmonella typhi* (Prem Anand et al. 1997). Furthermore, *Cypraea errones* crude methanol extract had stronger antibacterial and antifungal activity (Prem Anand and Edward 2002).

The majority of bacterial organisms can form biofilms on different surfaces in food production facilities and medical settings, leading to a persistent source of infections (Dongari-Bagtzoglou 2008; Percival et al. 2015; Galie et al. 2018; Flemming and Wuertz 2019). The communities of bacteria in the biofilm are held together by different biomolecules such as polysaccharides, secreted proteins, and extracellular DNA (Muhammad et al. 2020). Mollusc species that interact or live on surfaces manifested with biofilms have evolved means by which they prevent fouling of their shells by bacteria (Wahl et al. 2012). In the present investigation, the antibiofilm experiments showed that *Ts*-EtOAc has considerable biofilm inhibition activities against *S. aureus*, *P. aeruginosa*, *B. subtilis,* and *E. coli.* The highest biofilm inhibitory ratio was recorded against *S. aureus* (80%). Natural products from other marine organisms showed a considerable antibiofilm activities. For example, OctoPartenopin, a peptide extracted from the suckers of *Octopus vulgaris*, was used to synthesise four analogues that have proven functional in inhibiting the antibiofilm formation by *S. aureus*, *P. aeruginosa*, and *C. albicans* (Maselli et al. 2020). Moreover, the methanol/water tissue extracts of *Nerita albicilla* L. (Neritidae) and *N. oryzarum* Récluz (Neritidae) inhibited 93% and 95% of 40 different marine biofilm bacteria, respectively (Ramasamy and Murugan 2005).

### Cytotoxicity

Cancer is one of the most common causes of death worldwide (Bray et al. 2018). According to the International Agency for Research on Cancer (IARC), over 20 million new cancer cases are expected to be diagnosed in developing countries by 2025, most of them in low-and middle-income countries (Shah et al. 2019). Therefore, the search for new pharmaceuticals with distinct mechanisms of action against cancer is an urgent prerequisite. Chemical investigation of marine molluscs has resulted in isolating a broad spectrum of bioactive metabolites with anticancer properties (Ciavatta et al. 2017). Here we investigated the *in vitro* cytotoxic activity of *Ts*-EtOAc extract against three cancer cell lines: human kidney renal cell carcinoma UO-31, adenocarcinomic human alveolar basal epithelial cells A549, and human epidermoid carcinoma cells A431 representing kidney, lung, and skin cancers, respectively. These cancer types have a tremendous economic impact and a high mortality rate worldwide (Ljungberg et al. 2011; Siegel et al. 2018; Iqbal et al. 2019; Sung et al. 2021). The *Ts*-EtOAc extract was particularly chosen for cytotoxic investigation because it was the highest antioxidant extract among the *T. savignyi* extracts tested. Cancer initiation, progression, and resistance to therapy are all influenced by oxidative stress (Snezhkina et al. 2019). Natural antioxidants such as *Ts*-EtOAc may help inhibit tumour initiation by protecting DNA from oxidation and the resulting DNA damage caused by ROS, causing premalignant lesions to retreat and prevent them from progressing to cancer (Sithranga Boopathy and Kathiresan 2010). Indeed, *Ts*-EtOAc showed moderate *in vitro* toxicity towards UO-31, A549, and A431 cell lines. The best-observed effect was against UO-31 with an IC_50_ value of 19.96 ± 0.93 μg/mL compared to 5.165 ± 0.24 μg/mL for the control drug. However, the lowest effect was observed against the A431 cell line. These data suggest that *T. savignyi* can be used as an anticancer nutraceutical and, with proper modifications of extraction and purification, may lead to many anticancer drugs.

Other mollusc-derived compounds showed cytotoxic activities against different cancer cell lines. The isoquinoline alkaloid, jorumycin, from the nudibranch slug, *Jorunna funebris* Kelaart (Discodorididae), showed potent anticancer activity against lung adenocarcinoma cell line A549 with an IC_50_ = 12.5 ng/mL (Fontana et al. 2000). This compound also exhibited considerable cytotoxicity against other cell lines, including murine leukaemia cell line P388, human colon cancer cell line HT29, and human melanoma cell line MEL28 (Fontana et al. 2000; Saito et al. 2004). Zalypsis is another DNA-binding alkaloid isolated from *J. funebris* that shows potent antitumor activities against prostate, renal, breast, and colon cancers (Scott and Williams 2002; Chakraborty and Joy 2020). Kahalalide F is a cyclic depsipeptide isolated from the marine mollusc *E. rufescens* (Hamann and Scheuer 1993; Hamann et al. 1996). Kahalalide F was effective against several cell lines with solid multidrug resistance, such as human breast cancer cell lines H5578T and Hs-578T, human lung adenocarcinoma cell line A549 (IC_50_ = 0.135 μM) and other cell lines resistant to topoisomerase II inhibitors (Faircloth and Cuevas 2006). Serova et al. (2013) showed that elisidepsin (a synthetic derivative from kahalalide F) was effective against various human breast cancer cell lines, human colon cancer cell lines, human head and neck cancer cell lines, and human hepatocarcinoma (Serova et al. 2013). The depsipeptide kulokekahilide-2 from the sea slug, *Philinopsis speciosa* Pease (Aglajidae), was also efficient against murine leukaemia cell lines P338, human ovarian cancer cell line SK-OV-3, and human melanoma cell line MDA-MB-435 (Nakao et al. 2004).

### GC-MS analysis

The previous results of *Ts*-EtOAc extract bioactivity as an antioxidant, antimicrobial, and cytotoxic agent prompted additional chemical analysis to identify the chemical compound classes present in this extract. GC-MS analysis of *Ts*-EtOAc extract identified 45 compounds belonging to various chemical groups, including naphthalene, quinoline, alkane, alkyne, pyridazine, phenanthrene, cyclic ketones, cycloalkene, and aldehyde derivatives, ketones, monoterpenes, nitriles, fatty acid esters, stilbenoids, and sesquiterpenoids. Naphthalene derivatives were the most prevalent category, accounting for 25.57% of the total peak areas in the extract (95.82%). This is the first report of naphthalene derivatives in a marine gastropod. These metabolites could be produced due to the absorption of aromatic hydrocarbons by *T. savignyi*. The antioxidant, antimicrobial, and cytotoxic activities of *Ts*-EtOAc extract may be attributed to identified active metabolites. Previous literature data, for example, demonstrated that naphthalene is a cytotoxic moiety with a range of biological applications, including anticancer, antimicrobial, anti-inflammatory, and antiviral properties (Makar et al. 2019). Plant-derived naphthalene derivatives were reported to have DPPH radical scavenging activity as well as cytotoxic activity against cancer cell lines such as the human cholangiocarcinoma cancer cell line HuCCA-1, the lung adenocarcinoma cell line A549, the human hepatocellular liver carcinoma cell line HepG2, and the T-lymphoblast or acute lymphoblastic leukaemia cell line MOLT-3 (Molee et al. 2018). Furthermore, naphthalene derivatives from the endophytic fungus *Phomopsis fukushii* inhibited the growth of *S. aureus* (Li et al. 2021). A synthetic naphthalene compound, (*E*)-3-(2,3,4-trimethoxyphenyl)-1-(naphth-2-yl)-prop-2-en-1-one, inhibited the growth of four cancer cell lines: PC-3 human prostate cancer cell line, OVCAR human ovarian cancer cell line, IMR-32 human neuroblastoma cell line, and HEP-2 human hepatocellular liver carcinoma cell line (Budhiraja et al. 2012). Besides, the same compound demonstrated potent antimicrobial activity against four bacterial strains: *S. aureus*, *B. subtilis*, *P. aeruginosa*, *E. coli*, and *S. typhi*, and two fungal strains, *Aspergillus niger* and *C. albicans* (Budhiraja et al. 2012).

Furthermore, pyridazine derivatives have been reported to have diverse biological activities, including antiviral, anticancer, and antimicrobial properties (Butnariu and Mangalagiu 2009). Quinoline derivatives have diverse biological activities and constitute an important class of compounds for new drug development (Orhan Puskullu et al. 2013). Various synthetic quinoline derivatives were screened for their biological activities. Some derivatives showed antibacterial (Narender et al. 2006; Reddy et al. 2009; Matada et al. 2021), antifungal (Musiol et al. 2006), and cytotoxic activity (Costa et al. 2020). Phenanthrenes are a relatively small group of natural products derived primarily from plants. Almost all of the phenanthrene compounds isolated from plants demonstrated a variety of biological activities, including antioxidants (Behery et al. 2013; Woo et al. 2014), antimicrobial (Guo et al. 2016; Tóth et al. 2016), and cytotoxicity (Ma et al. 2016). Other chemical classes represented in *Ts*-EtOAc extract, such as ketones, monoterpenes, fatty acid esters, stilbenoids, and sesquiterpenoids, have been shown to have beneficial biological activities (Mallesha et al. 2012; Abdel-Aziz et al. 2018; Akinwumi et al. 2018; Mothana et al. 2018; Ghareeb et al. 2019; Elkhouly et al. 2020). Hamed et al. 2020;

### Molecular docking

Thymidylate kinase (TMK) is considered the key enzyme in the production of thymidine triphosphate and acts by catalysing the conversion of thymidine monophosphate into thymidine diphosphate (dTDP), which is subsequently phosphorylated by nucleoside diphosphate kinase to produce thymidine triphosphate (Hu et al. 2005; Cui et al. 2013). The thymidylate kinase enzyme plays a critical role in bacterial DNA biosynthesis. Therefore, TMK is a promising target for antibacterial drugs (Kawatkar et al. 2014). Indeed, TMK was validated as a potent antibacterial target for drugs designed to treat gram-positive bacterial infections (Keating et al. 2012). Docking studies using the MOE program (Barakat et al. 2018; Mustafa and Mostafa 2020) were carried out to predict the most suitable binding pose of identified compounds (**1–7**) in *Ts*-EtOAc extract using the bacterial enzyme; thymidylate kinase (PDB: 4QGG). The potencies of these identified compounds were evaluated computationally based on their docking scores (energy scores). This score represents the strength of the non-covalent interactions among numerous molecules within the binding pocket of a target protein. The higher the negative score is, the more beneficial interactions between the chemical and the target protein are. Most of the investigated compounds showed considerable binding interactions with important residues in the active site TMK (ID: 4QGG). However, compound **4** (2-[1-(4-cyano-1,2,3,4-tetrahydronaphthyl)] propanenitrile) showed the highest negative score of −9.117 kcal/mol based on performing binding interactions inside the active binding site via the formation of four hydrogen bonds with Arg36, Arg48, Arg92, and Gln37. Moreover, targeting the same bacterial enzyme, Barakat et al. (2018) found docking score of a synthetic pyrazole-dimedone derivative to be −6.86 kcal/mol through binding interactions with the amino groups of Arg70 and Gln101 and the crucial residues Phe66 and Arg92 of TMK. Moreover, Saminathan et al. (2020) docked two pyrazoline-thiocyanatoethanone derivatives; 1-(5-[4-fluorophenyl]-3-phenyl-4,5-dihydro-1H-pyrazol-1-yl)-2-thiocyanatoethanone (FSCN) and 1-(5-[4-chlorophenyl]-3-phenyl-4,5-dihydro-1H-pyrazol-1-yl)-2-thiocyanatoethanone (ClSCN) against the thymidylate kinase (ID: 4QGG). Their results showed that the two compounds formed different bonded interactions between compounds and TMK binding site residues with binding energies of −7.8 and −7.3 kJ/mol, respectively, for FSCN and ClSCN. The interactions of the FSCN involved six hydrogen bonds with the Arg36, Arg48, Arg92, and Thr16 residues of TMK, while the compound ClSCN interacted with protein 4QGG through five hydrogen bonds with the residues Arg36, Arg92, and Thr16 (Saminathan et al. 2020).

In the present investigation, the docking score of compound **4** is better than that of synthetic compounds and the standard drug ciprofloxacin, at 6.9 kcal/mol (Barakat et al. 2018). Thus, it can be a promising source of antibacterial drugs. The molecular docking results confirm those of *in vitro* antibacterial and antibiofilm analysis, where *Ts*-EtOAc showed better activities against *S. aureus* than other tested *T. savignyi* extracts.

## Conclusions

Marine molluscs represent an abundant source of metabolites with diverse biological activities. The preceding results suggest that the marine gastropod snail, *Thais savignyi*, is an excellent source of bioactive metabolites. The ethyl acetate extract from this snail possessed high antioxidant capacities and free radical scavenging activities. Moreover, the extract had considerable antimicrobial activity against pathogenic bacteria and fungi, and cytotoxicity against different cancer cell lines. Chemical analysis of the extract identified 45 compounds belonging to diverse classes of chemicals. The most abundant classes were naphthalene (predominant; 25.57%), pyridazine, quinoline alkane, and phenanthrene derivatives, nitriles, and fatty acid esters. Molecular docking results of the highly abundant compounds suggest their substantial potential to occupy the active sites of TMK, inhibiting the activity of this critical bacterial enzyme. These crucial interactions support the potential antibacterial activity of these compounds. In particular compound **4** (2-[1-(4-cyano-1,2,3,4-tetrahydronaphthyl)] propanenitrile), belongs to naphthalene derivatives, possesses a better drug-likeness nature and good inhibition behaviour with TMK protein. Therefore, more studies are needed to isolate active constituents of *Ts*-EtOAc extract for comprehensive drug discovery tests.
